# Psychometric Validation of the Gaming Disorder Scale for Adolescents (GADIS-A) in Dutch Among Flemish Adolescents

**DOI:** 10.5334/pb.1365

**Published:** 2025-07-02

**Authors:** Eva Grosemans, Rozane De Cock, Lowie Bradt, Huub Boonen, Bart Soenens

**Affiliations:** 1 Media Culture and Policy Lab, Faculty of Social Sciences, KU Leuven, Belgium; 2 Department of Developmental, Personality and Social Psychology, Faculty of Psychology and Education Sciences, Ghent University, Belgium; 3 Institute of Psychology, University of Lausanne, Lausanne, Switzerland; 4 Department of Special Needs Education, Ghent University, Ghent, Belgium; 5 CGG Integra-Limburg, Hasselt, Belgium

**Keywords:** gaming disorder, ICD-11, adolescents, screening, validation, addictive behavior

## Abstract

The Gaming Disorder Scale for Adolescents (GADIS-A, Paschke, Austermann & Thomasius, 2020) was the first screening tool for gaming disorder based on the new ICD-11 criteria. In order to increase the international applicability of the GADIS-A, the current study aimed to validate the psychometric properties of the Dutch version of the instrument. It was validated in a survey among 1773 Flemish (= Dutch-speaking part of Belgium) video game playing adolescents. EFA and CFA were performed to check the factor structure. The Video Game Addiction Test (VAT), gaming time, passion for gaming, Gaming Disorder Scale for Parents (GADIS-P), sensation seeking, impulsivity, adolescents’ school and social life, and simulated and monetary gambling were employed to derive construct validity, and life satisfaction, depression, and anxiety to check criterion validity. Analyses suggested a two-factor structure in the Dutch GADIS-A, similar to the original study: the first factor relates to negative consequences, while the second factor reveals cognitive-behavioral symptoms. Both subscales and the total scale showed acceptable-to-good internal consistency (*α* = 0.78-0.85). Significant correlations were established between GADIS-A and all other variables (except for sensation seeking), congruent with previous research. The Dutch version of the GADIS-A proved to be a reliable and valid tool for assessing gaming disorder in adolescents. It was also linked, for the first time, to the increasingly blurring lines between video gaming and monetary gambling.

## Introduction

Adolescents are avid video game players, with almost 90% of adolescents in Flanders (= Dutch-speaking part of Belgium) playing video games (Apestaartjaren, 2020). Video games can have a positive effect on players’ cognitive skills, such as improved task switching or processing speed (Nuyens et al., 2019), and can improve emotional and social skills (Granic, Lobel & Engels, 2014). They can even bring along eudaimonic experiences by increasing one’s feeling of relatedness, or by fostering thoughts about one’s own behavior and society in general (Daneels, Vandebosch & Walrave, 2020).

For some players, however, this seemingly innocent activity can turn into problematic use. The World Health Organization (WHO) has included video game addiction or gaming disorder in the 11th revision of the International Statistical Classification of Diseases and Related Health Problems (ICD-11), defining gaming disorder as:

“a pattern of gaming behavior […] characterized by impaired control over gaming, increasing priority given to gaming over other activities to the extent that gaming takes precedence over other interests and daily activities, and continuation or escalation of gaming despite the occurrence of negative consequences.”

To be classified as a disordered video game player, these three symptoms should be present for at least twelve months (WHO, 2022).

Moreover, the American Psychiatric Association (APA) added internet gaming disorder as a “condition requiring further study” in the DSM-5 (APA, 2013). One should experience at least five of nine criteria within a year to be classified as a disordered player. These symptoms are: (1) preoccupation with gaming, (2) withdrawal when not playing, (3) tolerance, (4) unsuccessful attempts to reduce or stop gaming, (5) giving up other activities, (6) continuation of gaming despite problems, (7) deceiving or covering up gaming, (8) gaming to escape adverse moods, and (9) risking or losing relationships or career opportunities due to excessive gaming. Previous research has found a gaming disorder prevalence (based on the DSM-5 criteria) between 1.2% and 5.9% in adolescents (Sugaya et al., 2019). Prevalence was higher amongst males (González-Bueso et al., 2018; Sugaya et al., 2019) and younger teenagers (André et al., 2020).

Associations have been found between gaming disorder and negative outcomes, such as aggression and emotional eating (Caner & Evgin, 2021), lower life satisfaction, depression, and loneliness (André et al., 2020; Colder Carras et al., 2017; Mentzoni et al., 2011), and anxiety (González-Bueso et al., 2018). Furthermore, gaming disorder has a negative impact on adolescents’ school life (Sugaya et al., 2019; Yu et al., 2019) and social life (Caner & Evgin, 2021). Lastly, gaming disorder is correlated with participation in gambling(-like) activities (Zendle, 2020), such as an increased spending on loot boxes (i.e., video game packages where the randomized content is hidden until opening them) (Rho et al., 2017).

Different screening tools have been used to measure gaming disorder in Dutch speaking countries. The Gaming Addiction Scale for Adolescents (Lemmens, Valkenburg & Peter, 2009), the Video Game Addiction Test (van Rooij et al., 2012), its successor the Clinical Video game Addiction Test 2.0 (van Rooij et al., 2017), and the Internet Gaming Disorder Scale (Lemmens, Valkenburg & Gentile, 2015) are widely validated and used. However, a validated screening instrument for adolescents based on the ICD-11 criteria was lacking, until the introduction of the Gaming Disorder Scale for Adolescents (GADIS-A) scale by Paschke, Austermann, and Thomasius (2020). A recent review (Saunders et al., 2025) has listed several other instruments that have been developed since then, such as the Gaming Disorder Test (GDT, Pontes et al., 2021), the Gaming Disorder Symptom Questionnaire 21 Items (GDSQ-21, Zhang et al., 2022), the Gaming Engagement Screener test (GAMES test, Hong et al., 2023), the Lee Morrell Gaming Disorder Questionnaire (Lee et al., 2022), and the Gaming Disorder Identification Test (GADIT, Chan et al., 2024).

The GADIS-A was developed and validated on a sample of German adolescents, proving to measure gaming disorder reliably. Based on the new ICD-11 criteria, it contains two subscales: negative consequences and cognitive-behavioral symptoms. The latter factor reflects easily observable symptoms, such as the inability to stop gaming or the neglect of daily duties, whereas the former reflects long-term negative consequences, such as the loss of personal relationships. The subscales are complemented by a timing question accounting for long-term consequences of gaming disorder. In the German sample, 3.7% of the video game playing respondents met all cut-off scores, classifying them as disordered gamers. In 2021, the Gaming Disorder Scale for Parents (GADIS-P) was introduced, enabling the assessment of gaming disorder in adolescents using parental ratings (Paschke, Austermann & Thomasius, 2021).

To make the GADIS-A more widely applicable, we translated the GADIS-A into Dutch and validated it on a sample of Flemish adolescents in a large-scale survey. The scale is tested against other video game measures, personality traits and mental health outcomes, social and school life measures, and lastly, gambling activities. To the authors’ knowledge, this is the first time the GADIS-A is used in Dutch. Moreover, this is the first time a Dutch gaming disorder scale is related to simulated gambling elements, such as loot boxes, and gambling among adolescents.

We propose the following hypotheses:

H1: The Dutch version of GADIS-A will show a similar two-factor structure as the German version.

H2: The reliability of the scale will be at least in acceptable range (*α* > 0.7).

H3: The Dutch version of GADIS-A will show significant correlations with video gaming, personality traits, social life, school life, gambling, and mental health outcomes.

## Methods

### Procedure

To validate the GADIS-A scale in Dutch, a survey was distributed in 13 Flemish high schools between November 2021 and February 2022. The survey included questions about video gaming, simulated gambling, monetary gambling, mental health outcomes and personality traits. Adolescents took part online or on paper, either during class time or in their free time. Parents were invited to take part in the study online through the schools’ mailing system.

Three different versions of the adolescent survey were used to reduce completion time. Respondents were divided across the three versions equally, at random. All versions included questions on video gaming, mental health outcomes, and personality traits, as well as socio-demographic questions (such as gender and age). Next to that, the GADIS-A was included in the core set of each version of the survey. The remaining part of the survey contained additional measures, focusing on either (1) video gaming (version A), (2) personality traits (version B), or (3) persuasive media content (version C; not used in this particular results presentation). Next to that, parents filled out a similar survey.

### Participants

In total, 2443 adolescents and 823 parents took part in the study. Only adolescents who filled out all GADIS-A items were analyzed, resulting in 1773 respondents (*M* age = 13.98, *SD* = 1.48). 52.5% of the final sample identified themselves as male, 45.9% as female, and 1.5% as “other” or left the question blank. Only parents who could be matched to their child and who completed the GADIS-P (and of whom their child had completed the GADIS-A) were considered, resulting in 147 parent-child dyads. Parents’ mean age was 44.71 years (*SD* = 5.82). 78.8% of parents identified themselves as female, 21.2% as male (one parent left this gender question blank). Children in these parent-child dyads mostly identified themselves as male (66.0%, 32.7% female, 1.4% other).

### Measures

A variety of measures were employed to validate the usability and validity of the Dutch version of the GADIS-A. Table 1 describes the used measures, their n, the corresponding version of the survey in which the variables were used, and Cronbach’s *α* and McDonald’s *ω* for each scale.

**Table 1:** Descriptive statistics of the used variables


NAME	*N*	SURVEY VERSION*	*α*	*ω*

**GADIS-A**	1773	Core	0.85	0.85

**Negative consequences**			*0.78*	*0.78*

**Cognitive-behavioral symptoms**			*0.78*	*0.78*

**Temporal item**			*/*	*/*

**VAT**	580	A	0.91	0.91

**GADIS-P**	147	Parental survey	0.91	0.91

**Gaming time (weekday and weekend day)**	1773	Core	/	/

**Harmonious vs. Obsessive passion for gaming**	1755	Core		

**Harmonious passion for gaming**			*0.71*	*0.78*

**Obsessive passion for gaming**			*0.84*	*0.84*

**Sensation Seeking**	533	B	0.80	0.81

**Impulsivity**	529	B	0.77	0.76

**Life Satisfaction**	1640	Core	0.86	0.86

**Depression**	539	B	0.87	0.87

**Anxiety**	535	B	0.78	0.76

**Support from family**	1663	Core	/	/

**Support from friends**	1663	Core	/	/

**Sense of belonging to an online community**	1663	Core	/	/

**Schooling attitude**	1773	Core	/	/

**School grades**	1773	Core	/	/

**Participation in simulated gambling activities**	1617	Core	0.80	0.80

**Participation in online gambling activities**	1584	Core	0.95	0.95

**Monetary gambling intention**	1578	Core	/	/


Notes: *Refers to the version of the survey in which the variable was included: - “Core” = this variable was included in the core set of questions, in all adolescent versions of the survey. - “A” = this variable was included in the version of the survey that focused on additional measures of video gaming. - “B” = this variable was included in the version of the adolescent survey that focused on additional measures of personality traits. - “Parental survey” = this variable was included in the parental survey.

#### Gaming disorder

##### Gaming Disorder Scale for Adolescents (GADIS-A)

The Gaming Disorder Scale for Adolescents (GADIS-A) was translated to Dutch, to validate it in a population of Flemish adolescents. It comprises nine items, plus an item on the frequency of symptoms. The first nine items are divided into two subscales: five items regarding negative consequences, and four items regarding cognitive-behavioral symptoms. These nine items are answered using five response options (ranging from 0 = “strongly disagree”, to 4 = “strongly agree”), resulting in a total score between 0 and 36. The timing question is answered on a four-point scale (ranging from 0 = “not at all”, to 3 = “nearly daily”). The English version and its Dutch translation can be found in Table 2.

**Table 2:** English and Dutch version of the GADIS-A.


NR	ENGLISH	DUTCH

**1**	I often play games more frequently and longer than I planned to or agreed upon with my parents.	Ik game vaak meer en langer dan ik van plan was of met mijn ouders had afgesproken.

**2**	I often cannot stop gaming even though it would be sensible to do so or for example my parents have told me to stop.	Ik kan vaak niet stoppen met gamen, ook al zou het verstandig zijn om dat te doen of hebben mijn ouders gezegd dat ik moet stoppen.

**3**	I often do not pursue interests outside the digital world (e.g., meeting friends or partner in real life, attending sports clubs/societies, reading books, making music) because I prefer gaming.	Ik heb vaak geen interesses buiten de digitale wereld (bv. afspreken met vrienden in het echte leven, naar (sport)clubs gaan, boeken lezen, muziek maken) omdat ik liever game.

**4**	I neglect daily duties (e.g., grocery shopping, cleaning, tidying up after myself, tidying my room, obligations for school/apprenticeship/job) because I prefer gaming.	Ik verwaarloos dagelijkse taken (bv. boodschappen doen, schoonmaken, opruimen, verplichtingen voor school/stage/job) omdat ik liever game.

**5**	I often continue gaming even though it causes me stress with others (e.g., my parents, siblings, friends, partner, teachers).	Ik ga vaak door met gamen, ook al veroorzaakt het stress bij anderen (bv. bij mijn ouders, broers en zussen, vrienden, partner, leraren).

**6**	I continue gaming although it harms my performance at school/apprenticeship/job (e.g., by being late, not participating in class, neglecting homework, worse grades).	Ik ga door met gamen, hoewel het mijn prestaties op school/stage/job beïnvloedt (bv. door te laat te komen, niet deel te nemen aan de les, huiswerk te verwaarlozen, slechtere cijfers).

**7**	Due to gaming, I neglect my appearance, my personal hygiene, and/or my health (e.g., sleep, nutrition, exercise).	Door het gamen verwaarloos ik mijn uiterlijk, mijn persoonlijke hygiëne en/of mijn gezondheid (bv. met betrekking tot slaap, voeding, lichaamsbeweging).

**8**	Due to gaming, I risk losing important relationships (friends, family, partner) or have lost them already.	Door het gamen loop ik het risico belangrijke relaties (vrienden, familie, partner) te verliezen of ben ik ze al kwijt.

**9**	Due to gaming I have disadvantages at school/apprenticeship/job (e.g., bad [final] grades, inability to continue to the next grade/no graduation, no apprenticeship or university spot, poor reference, warning/dismissal).	Vanwege het gamen ondervind ik nadelen op mijn school/stage/job (bv. slechte punten, blijven zitten, waarschuwing/ontslag).

**10**	How often did you experience such problems, conflicts, or difficulties due to gaming during the past year? Did this only occur on single days, during longer periods of several days to weeks, or was it almost daily?	Hoe vaak ondervond je het afgelopen jaar problemen, conflicten of moeilijkheden door gamen?


*Items 1, 2, 4 and 5 = cognitive-behavioral symptoms (second subscale)*. *Items 3, 6, 7, 8 and 9 = negative consequences (first subscale)*. *Item 10 = temporal issue*.

##### Video Game Addiction Test (VAT)

To assure the construct validity of the GADIS-A, the Video Game Addiction Test (VAT) was used (van Rooij et al., 2012). The VAT was developed and validated on a sample of Dutch teenagers (ages 13–16 years) in The Netherlands. Therefore, it was deemed usable in our own sample, considering our respondents’ mother tongue is Dutch as well. It consists of 14 items, with five Likert-scale response options (ranging from “never” to “very often”). An example item was “How often do you game to forget about problems?”.

##### Gaming Disorder Scale for Parents (GADIS-P)

The Gaming Disorder Scale for Parents (GADIS-P) (Paschke, Austermann & Thomasius, 2021) contains the same items as the GADIS-A but is completed by parents who rate their child’s gaming behavior. The first nine items are answered using five Likert-scale response options (ranging from 0 = “strongly disagree”, to 4 = “strongly agree”), resulting in a total score between 0 and 36. Example items included “My child often plays games more frequently and longer than he/she planned to or agreed upon with me or my partner” and “Due to gaming, my child neglects his/her appearance, personal hygiene, and/or health (e.g., sleep, nutrition, exercise)”. The timing question is answered using a four-point scale (ranging from 0 = “not at all”, to 3 = “nearly daily”).

#### Time spent playing video games and passion for gaming

Time spent playing video games during school days and weekend days was measured in hours. Next, types of engagement in video game play were measured (Przybylski et al., 2009). The Harmonious vs Obsessive Passion for Gaming scale consists of 10 items (five assessing harmonious passion, five assessing obsessive passion), using five Likert-scale response options (ranging from “not at all” to “totally”). Example items included “Playing video games allows me to live memorable experiences” (harmonious passion) and “I cannot live without playing video games” (obsessive passion).

#### Personality traits

Sensation Seeking was measured using the Brief Sensation Seeking Scale (Hoyle et al., 2002). It consists of eight items, using five Likert-scale response items. The scale results in an average score between 1 and 5, with higher scores indicating higher levels of sensation seeking. Impulsivity was measured using the Barratt Impulsiveness Scale–Brief (BIS-Brief) (Steinberg et al., 2013). The BIS-Brief consists of eight items using four Likert-scale response items, resulting in an average score between 1 and 4, with higher scores indicating higher levels of impulsivity.

#### Social life

Support from family, support from friends, and online belonging were each measured using one item (“How often do you feel that you get support from your family when you need it?”, “How often do you feel that you get support from your friends when you need it?”, and “How strongly do you feel you belong to an online community?”). Higher scores indicated higher levels of support and a higher feeling of belonging.

#### School life

School life was assessed using two items, measuring respondents’ academic performance (“How are things going at school in terms of grades?”) and attitude towards school (“How much do you like going to school?”). Scores ranged between 1 and 10, with higher scores indicating a better performance at school and a more positive attitude towards school.

#### Simulated gambling and monetary gambling

Both simulated gambling (i.e., gambling(-like) activities in and around video games) and monetary gambling frequency were assessed. Seven items were used to measure simulated gambling: opening free loot boxes, buying loot boxes using real money, selling items from loot boxes, spinning a wheel in order to win a prize, buying items in social casino games, watching loot box openings, and watching gambling videos. Moreover, six online monetary gambling activities were used: online betting, online sports betting, online games with monetary prizes, online poker in order to win money, online games organized by the National Lottery, and online lottery games. Respondents indicated how often they had participated in each of these activities in the past 12 months, using a seven Likert-type scale. This resulted in an average total score between 1 and 7 for both simulated and monetary gambling, with higher scores indicating more frequent participation. Lastly, monetary gambling intention was assessed using a single item.

#### Mental health outcomes

Life satisfaction was measured using the Satisfaction With Life Scale (Diener et al., 1985). It consists of five items using five Likert-scale response options, resulting in a total score between 5 and 25. The higher the score, the more satisfied with life one is. Depression and anxiety were each measured by six items, using four Likert-scale response options. Depression was measured by the CES-D6 (Center for Epidemiologic Studies Depression scale) (Radloff, 1977), and anxiety by the STAI-6 (Six-Item State Anxiety Scale) (Marteau & Bekker, 1992). Both scales result in a total score between 0 and 18, with higher scores indicating higher feelings of depression or anxiety.

### Statistical analysis

Skewness (absolute value of > 2.0 for substantial non-normality) and kurtosis (> 7.0) were calculated to test for the normality distribution (Kim, 2013). Exploratory Factor Analysis (EFA) was executed on the Pearson correlation matrix in R in order to determine the usability of the proposed two-factor structure (Paschke, Austermann & Thomasius, 2020). The Kaiser-Meyer-Olkin (KMO) criterion and Bartlett’s Test of Sphericity were used to assess suitability for factor analysis. Next, confirmatory factor analysis (CFA) was performed using lavaan in R (Rosseel, 2012), based on the covariance matrix derived from Pearson correlations among the standardized observed variables. Goodness of fit was evaluated using the chi-square value, the standardized root mean squared residual (SRMR), the root mean square error of approximation (RMSEA), the Tucker-Lewis Index (TLI), and the root mean square residual (RMSR). Lastly, the comparative fit (CFI) was calculated. We employed the following criteria to determine model goodness of fit: RMSR ≤ 0.05, and following the original GADIS-A study (Paschke, Austermann & Thomasius, 2020): RMSEA < 0.1, SRMR < 0.08, TLI ≥ 0.95, CFI ≥ 0.95. Sum scores were calculated for each factor scale.

Internal consistency was assessed using Cronbach’s α and McDonald’s ω, with scores ≥ 0.70 deemed acceptable, ≥ 0.80 deemed good, and ≥ 0.90 deemed excellent (Nunnally & Bernstein, 1994). To assure the construct validity of the GADIS-A, Pearson and Spearman rank correlations with video gaming, personality traits, social life and school life, and gambling were assessed. To test the criterion validity of the GADIS-A, partial correlations with mental health outcomes were checked, whilst controlling for gender and age. For these correlations, the following criteria were used: r ≤ 0.29 for weak correlations, 0.30 ≥ r ≤ 0.49 for moderate correlations, and r ≥ 0.50 for large correlations (Cohen, 1988).

Descriptive numbers regarding GADIS-A and GADIS-P scores are provided, including gender differences for the total GADIS-A and both subscales. Because of the low number of respondents in the “other” or missing category, only differences between boys and girls are computed. The cut-off scores, proposed by Paschke, Austermann, and Thomasius, (2020), will be employed to determine the prevalence of gaming disorder in our sample. These cut-off scores include a score of ≥ 12 on the total GADIS-A scale, a score of > 5 on the first subscale (negative consequences), a score of > 9 on the second subscale (cognitive behavioral symptoms), and experiencing problems at least for longer periods or daily.

### Ethics

Parents and legal caretakers of the students in participating schools were informed about the study via mail, prior to the start of the survey, and gave passive consent for their child’s participation. Only students who were not deregistered were presented with the survey. Respondents were asked to agree to an informed consent before continuing with the survey. This informed consent stated that participation was completely voluntary, and that all responses remained confidential. Respondents were able to stop their participation, without consequences. The procedure received ethical approval via the Social and Societal Ethics Committee at KU Leuven (G-2021-3439-R2(AMD)), and has been performed in accordance with the Declaration of Helsinki.

## Results

### Factor structure

KMO was 0.88, with Bartlett’s Test of Sphericity being significant (*p* < 0.001), meaning the data was suitable for factorial analyses.

Based on the scree plot, the Kaiser criterion (eigenvalues > 1), and Parallel Analysis (Patil et al., 2017), a two-factor solution was proposed. Because we expected the two factors to be correlated, we opted for a promax rotation, using the Minimum Residual (MINRES) method. The eigenvalue was 2.33 for the first factor and 1.97 for the second factor, with communalities ranging from 0.43 to 0.57. One item (item 3) showed a rather low communality of 0.20. However, since the item-total correlation of this item was 0.38, it was retained in the scale. The proposed two factors explained a total variance of 0.48 (variance of factor one = 0.26, factor two = 0.22). Factor loadings ranged between 0.32 and 0.84 for factor one and 0.36 and 0.85 for factor two. Item 4 had similar loadings on both factors (0.39 on factor one and 0.36 on factor two). Here, we followed the reasoning of the original scale, including it in the second factor (Paschke et al., 2020). The model demonstrated good fit (empirical *χ^2^*(19) = 150.86, *p* < 0.001, RMSEA = 0.089, RMSR = 0.030, TLI = 0.910).

**Figure 1:**
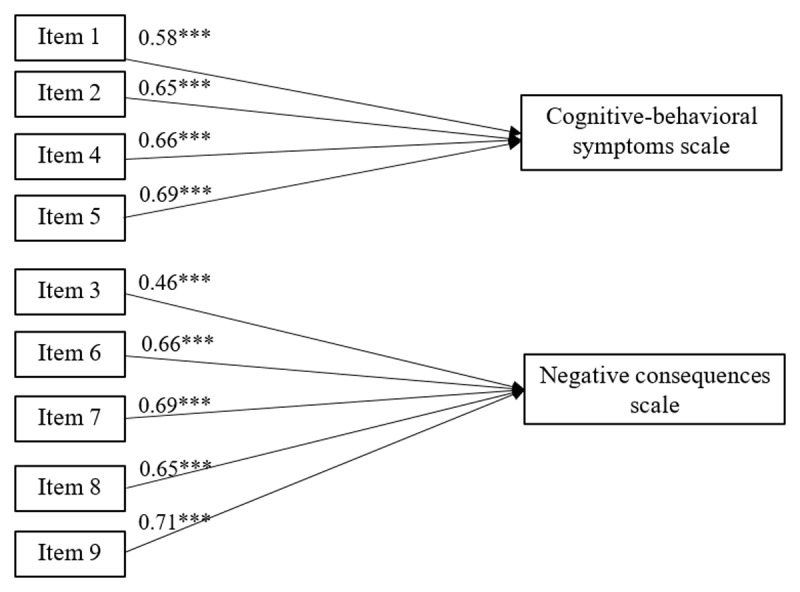
CFA factor loadings. *** p < .001.

CFA models were estimated using maximum likelihood (ML) estimation. CFA applying the two-factor solution revealed a good fit to the data (standard *χ^2^*(22) = 271.29, *p* < 0.001, RMSEA = 0.078, SRMR = 0.040, CFI = 0.957, TLI = 0.929). Item 1 & 2, 4 & 6, 4 & 9 and 6 & 9 were given error correlations in the model, because they used similar expressions. A confirmatory two-factor model with the higher order factor was not identified because the two factors were too highly correlated. Figure 1 describes the CFA-factor loadings for both factors.

In conclusion, the same two-factor model from the original article (Paschke, Austermann & Thomasius, 2020) emerged from our sample, with five items mirroring the negative consequences dimension (item 3, 6, 7, 8, and 9), and four items reflecting the cognitive-behavioral symptoms (item 1, 2, 4, and 5). EFA- and CFA-factor loadings are presented in Table 3.

**Table 3:** EFA- and CFA-factor loadings.


GADIS-A ITEM	FACTOR 1	FACTOR 2	COMMUNALITIES

**Item 1 EFA**	–0.18	**0.85**	0.54

**Item 1 CFA**		0.58	

**Item 2 EFA**	–0.04	**0.79**	0.57

**Item 2 CFA**		0.65	

**Item 3 EFA**	**0.32**	0.11	0.20

**Item 3 CFA**	0.46		

**Item 4 EFA**	0.39	**0.36**	0.48

**Item 4 CFA**		0.66	

**Item 5 EFA**	0.19	**0.51**	0.43

**Item 5 CFA**		0.69	

**Item 6 EFA**	**0.49**	0.30	0.53

**Item 6 CFA**	0.66		

**Item 7 EFA**	**0.72**	–0.04	0.47

**Item 7 CFA**	0.69		

**Item 8 EFA**	**0.84**	–0.21	0.50

**Item 8 CFA**	0.65		

**Item 9 EFA**	**0.70**	0.08	0.57

**Item 9 CFA**	0.71		


*GADIS-A factor 1 = negative consequences (= items 3, 6, 7, 8, 9)*. *GADIS-A factor 2 = cognitive-behavioral symptoms (= items 1, 2, 4, 5)*.

To test for scalar invariance, the model with equal loadings and intercepts was compared to the unconstrained model. The CFI dropped from 0.95 to 0.93 (ΔCFI = 0.02), exceeding the recommended 0.01 threshold (Cheung & Rensvold, 2002). This suggests that scalar invariance does not hold, and comparisons of observed means between boys and girls may be biased. However, further inspection revealed that two items in particular (item 1 and item 2) did not display scalar invariance. When unconstraining the intercepts and factor loadings of these two items (thereby keeping the intercepts and loadings of all other items constrained), the fit of the model did not differ significantly from the fully unconstrained model (ΔCFI < 0.01). So, with the exception of only two items, there was evidence for scalar invariance across gender.

### Internal consistency

For the total GADIS-A scale (nine items without the temporal issue), a Cronbach’s *α* of 0.85 and a McDonald’s *ω* of 0.85 were measured, showing good internal consistency. The first subscale showed an acceptable Cronbach’s *α* of 0.78 and a McDonald’s *ω* of 0.78. The second subscale also showed an acceptable Cronbach’s *α* of 0.78 and a McDonald’s *ω* of 0.78 (see also Table 1).

### Construct validity

All items and scales were distributed normally, except for gaming time (school days and weekend days) and online monetary gambling frequency. Table 4 shows the bivariate correlations between the different items and scales and total GADIS-A score. Correlations for the two subscales (negative consequences and cognitive-behavioral symptoms) were similar to correlations with the total GADIS-A score, which is why only the latter are reported here. This also coincides with the strategy used in the original GADIS-A scale development (Paschke, Austermann & Thomasius, 2020).

**Table 4:** Bivariate correlations.


VARIABLE	GADIS-A TOTAL SCORE (*R/ρ*)

**VAT total**	0.744*** (*r*)

**Gaming time school day**	0.415*** (*ρ*)

**Gaming time weekend day**	0.479*** (*ρ*)

**Harmonious passion for gaming**	0.413*** (*r*)

**Obsessive passion for gaming**	0.697*** (*r*)

**Sensation seeking**	0.059 (ns) (*r*)

**Impulsivity**	0.250*** (*r*)

**Support from family**	–0.137*** (*r*)

**Support from friends**	–0.116*** (*r*)

**Online belonging**	0.170*** (*r*)

**Attitude towards school**	–0.216*** (*r*)

**School performance**	–0.233*** (*r*)

**Simulated gambling frequency**	0.404*** (*r*)

**Online monetary gambling frequency**	0.266*** (*ρ*)

**Monetary gambling intention**	0.185*** (*r*)

**GADIS-P (parent-child dyads)**	0.381*** (*r*)


*r = Pearson’s r*, ρ = Spearman’s rho, ***p < 0.001, (ns) = not significant.

#### Video gaming

Analyses revealed a strong positive correlation between the total score on the GADIS-A and the score on the VAT (*r* = 0.74). Correlations between total GADIS-A score and gaming time were positive and moderate in size (school day: *ρ* = 0.42; weekend day: *ρ* = 0.48). Lastly, both harmonious (*r* = 0.41) and obsessive (*r* = 0.70) passion for gaming were moderately and strongly correlated with total GADIS-A score. All correlations were significant (*p* < 0.001).

#### Personality traits

Impulsivity (*r* = 0.25, *p* < 0.001) was correlated positively with total GADIS-A score, albeit weakly, while sensation seeking had no significant correlation with the total GADIS-A score (*p* = 0.18).

#### Social life and school life

Both support from family (*r* = –0.14) and from friends (*r* = –0.12) correlated negatively with total GADIS-A score, while online belonging correlated positively (*r* = 0.17). Attitude towards school (*r* = –0.22) and performance at school (*r* = –0.23) correlated negatively with total GADIS-A score. All correlations were significant (*p* < 0.001), albeit only small.

#### Gambling

All gambling items correlated significantly with total GADIS-A score (*p* < 0.001). Simulated gambling participation correlated positively and moderately (*r* = 0.40) with total GADIS-A score, just as online monetary gambling participation (*ρ* = 0.27) and monetary gambling intention (*r* = 0.19). The last two correlations were, however, small.

#### GADIS-P

Lastly, we compared parent and child assessments of disordered video game play by looking at total scores on the GADIS-A and GADIS-P for 147 parent-child dyads. Both scales were positively and moderately correlated (*r* = 0.38, *p* < 0.001).

### Criterion validity

Regarding mental health outcomes, we checked for partial correlations, whilst controlling for gender and age. Life satisfaction correlated negatively with total GADIS-A score (*r* = –0.25, *p* < 0.001), while depression (*r* = 0.24, *p* < 0.001) and anxiety (*r* = 0.18, *p* < 0.001) correlated positively. These correlations were small in size.

### Data

On average, respondents scored 7.88 on the GADIS-A (*SD* = 6.33). Boys (*M* = 9.40, *SD* = 6.27) scored significantly higher than girls (*M* = 6.02, *SD* = 5.72) (t(1732) = 11.68, *p* < 0.001; Cohen’s *d* = 0.562). Age was negatively correlated with total GADIS-A score, albeit very weakly (*r* = –0.097, *p* < 0.001). The average score on the GADIS-P was 10.25 (*SD* = 7.03).

Mean score on the first subscale, negative consequences, was 3.12 (*SD* = 3.43). Boys scored significantly higher than girls (t(1727.31) = 10.29, *p* < 0.001; Cohen’s *d* = 0.489). Mean score on the second subscale, cognitive-behavioral symptoms, was 4.76 (*SD* = 3.55). Again, boys scored significantly higher than girls (t(1732) = 10.85, *p* < 0.001; Cohen’s *d* = 0.522). Lastly, mean score on the temporal item was 0.49 (*SD* = 0.62), with boys scoring significantly higher than girls (t(1727.05) = 10.40, *p* < 0.001; Cohen’s *d* = 0.494). All scores are shown in Table 5.

**Table 5:** Mean and SD for GADIS-A and GADIS-P, including gender differences.


ITEM	SAMPLE	*M*	*SD*

**GADIS-A total**	Full sample	7.88	6.33

	Boys	9.40	6.27

	Girls	6.02	5.72

**Negative consequences**	Full sample	3.12	3.43

	Boys	3.84	3.54

	Girls	2.23	2.94

**Cognitive behavioral symptoms**	Full sample	4.76	3.55

	Boys	5.56	3.44

	Girls	3.78	3.38

**Temporal item**	Full sample	0.49	0.62

	Boys	0.63	0.65

	Girls	0.33	0.54

**GADIS-P (parent-child dyads)**		10.25	7.03


#### Gaming disorder

Following the cut-off criteria by Paschke, Austermann, and Thomasius (2020), 2.0% of our sample (*n* = 35) was labelled as gaming disordered. On average, these disordered gamers were 13.54 years old (*SD* = 1.20) and mainly identified themselves as male (60.0%). They spent 2.57 hours per weekday playing video games (*SD* = 1.97), and 5.69 hours per weekend day (*SD* = 3.83). Table 6 provides the gender split for gaming disorder. Due to the low number of respondents in the disordered video game players group, no statements can be made regarding the differences between disordered and non-disordered gamers.

**Table 6:** Gender split for disordered video game players.


GENDER	*n*	%

Male	21	60.0

Female	12	34.3

Missing gender	2	5.7

TOTAL	35	100


## Discussion

The aim of this study was to scrutinize the psychometric properties of the Dutch version of the Gaming Disorder Scale for Adolescents (GADIS-A) (Paschke, Austermann & Thomasius, 2020), by using a large-scale survey amongst over 1700 Flemish adolescents. First, EFA and CFA suggested two factors (negative consequences and cognitive-behavioral symptoms), explaining 48% of the variance. This is slightly less compared to the original scale of Paschke, Austermann, and Thomasius (2020), which explained 65% of the variance using the same two-factor structure. Next, the scale proved to be reliable, with a Cronbach’s *α* of 0.85 for the total scale, and 0.78 for both subscales. Although slightly less than the original scale, it still showed acceptable to good internal consistency.

The Dutch version of the GADIS-A proved to be a valid scale to measure gaming disorder in adolescents. It showed significant positive correlations with other video gaming related measures, such as the Video Game Addiction Test (VAT), time spent playing video games (congruent with previous studies, such as Pontes et al., 2022; Rho et al., 2017), and harmonious and obsessive passion for gaming. In line with what could be expected based on previous studies on problematic gaming, the GADIS-A was positively correlated with impulsivity (Rho et al., 2017) and online belonging (Colder Carras et al., 2017), and negatively correlated with support from family and friends (André et al., 2020) and teenagers’ school life (Sugaya et al., 2019; Yu et al., 2019). Gambling (both simulated and monetary) was significantly positively correlated with total score on the GADIS-A, similar to previous research (Zendle, 2020). In contrast with research by Chiu, Lee, and Huang (2004), but congruent with other studies (Collins, Freeman & Chamarro-Premuzic, 2012; Walther, Morgenstern & Hanewinkel, 2012), sensation seeking was not significantly correlated with the GADIS-A score. Lastly, the GADIS-A score showed good criterion validity towards mental health outcomes (positive for anxiety and depression, negative for life satisfaction). This finding tallies with previous findings (Mentzoni et al., 2011; Sugaya et al., 2019). Using 147 parent-child dyads, we could conclude that the total score on the GADIS-A was significantly correlated with the total GADIS-P score as well. This was in accordance with the original study on the GADIS-P (Paschke, Austermann & Thomasius, 2021), although in this study, the correlations were larger.

Boys scored significantly higher than girls on the total GADIS-A. Following the cut-off criteria, 2.0% of our sample was labelled as gaming disordered. This was comparable to the prevalence of 3.7%, found in the original GADIS-A study (Paschke, Austermann & Thomasius, 2020), as well as to the prevalence in studies using a variety of gaming addiction scales, such as a prevalence of around 2% using the Game Addiction Scale (André et al., 2020) and the Gaming Disorder Test (Pontes et al., 2022). This study therefore adds to the robustness of the GADIS-A and the ICD-11 framework as a measure of disordered video gaming prevalence (Pontes et al., 2022). On average, these disordered gamers were 13.54 years old, and most of them identified themselves as male (60.0%), similar to previous studies (González-Bueso et al., 2018; Sugaya et al., 2019). They spent over two and a half hours per weekday playing video games, and over five and a half hours per weekend day.

The validation of the GADIS-A in a different context, such as Flanders, offers a number of significant benefits. It broadens the applicability of the scale across different studies and contexts, enhancing the generalizability of findings and strengthening the robustness of research on video gaming and, more broadly, internet disorders. Additionally, validation can identify potential biases or limitations in the original scale that may not be apparent in the original population but could emerge in a different cultural or linguistic context. Furthermore, by validating the scale across diverse cultural contexts, we contribute to the understanding of cultural diversity in psychological research and practice.

### Limitations and future research

Only a small number of respondents in our sample reached the cut-off score for gaming disorder, making it hard to make statements about this group or compare it to non-disordered video game players. Therefore, future research could validate the GADIS-A in a sample of heavy or disordered video game players. While we only made a distinction between disordered and non-disordered video game players, future studies could focus on highly frequent gamers who may be at risk as well. Moreover, since the translation of the GADIS-A is the first scale in Dutch language to use the ICD-11 criteria for diagnosing Gaming Disorder, the only way to compare it with a validated scale was to use a formerly used scale based on DSM 5 criteria, which differs from the ICD-11 criteria. Future studies with gaming disordered adolescents could include a comparison of the GADIS-A scores against semi-structured clinical interviews. Next, similar to the original paper by Paschke, Austermann, and Thomasius (2020), some items of the GADIS-A were intercorrelated. This was mainly due to the similar wording of some of the items (such as the use of “neglect” in both item 4 and 6). These correlations should not be taken lightly, and future research could benefit from slightly rewriting the items, in order to avoid such correlations. While the GADIS-A proposes a two-factor structure for disordered video game play, this structure does not directly map onto the three core diagnostic criteria of the ICD-11 conceptual framework. Moreover, some of the labels are not entirely consistent with the content of the items (e.g., item 3 (loss of interest in other activities), classified under “negative consequences”, more directly reflects Criterion B (“increasing priority”)). Future research could therefore reevaluate both the factor structure and the labels of the proposed two-factor model. On top of that, since the same sample was used for both EFA and CFA, additional studies are needed to accumulate further evidence of validity, as a single study cannot serve as sufficient justification for the validity of the questionnaire. Next, our results pointed towards a positive correlation between parental and adolescent ratings of disordered video game play. While this correlation implies some level of agreement or shared perception between parents and children regarding these traits, it could potentially be influenced by common environmental, behavioral, or genetic factors. Therefore, further analysis and contextualization are needed to understand the meaning of this correlation for the parent-child relationship. Lastly, based on the only moderate correlation between GADIS-A and GADIS-P scores, the results of future studies in the Flemish population may be dependent on the informant; the usage of multiple informants could thus strengthen the reliability of these studies.

## Conclusion

This study tested the psychometric validity of the Dutch version of the Gaming Disorder Scale for Adolescents (GADIS-A) (Paschke, Austermann & Thomasius, 2020). Using a large-scale survey amongst Flemish adolescents and their parents, the Dutch GADIS-A proved to be a reliable and valid tool in measuring gaming disorder in video game playing adolescents. In our sample, around 2% of respondents was labelled as a disordered gamer, of whom most identified themselves as male. Increased scores on the GADIS-A were correlated with: increased gaming time and a higher obsessive passion for gaming; higher levels of impulsivity; less support from family and friends but a higher sense of online belonging; lower life satisfaction and increased feelings of depression and anxiety; less academic liking and performance; and increased participation in gambling activities. Overall, these results show that the Dutch version of the GADIS-A can be a useful scale to assess gaming disorder in adolescents, according to the new ICD-11 criteria. This contributes to the accumulating evidence of validity for the GADIS-A. To the authors’ knowledge, this is the first time the GADIS-A was validated and used in Dutch.

## Data Accessibility Statement

The anonymized dataset (in Dutch) and the corresponding codebook (in English) are accessible via this anonymized OSF-link.
